# A scoping review of risk-stratified bowel screening: current evidence, future directions

**DOI:** 10.1007/s10552-022-01568-9

**Published:** 2022-03-20

**Authors:** J. M. Cairns, S. Greenley, O. Bamidele, D. Weller

**Affiliations:** 1 Hull York Medical School, University of Hull, Cottingham Road, Hull, HU6 7HR UK; 2 Centre for Population Health Sciences, University of Edinburgh, Teviot Place, Edinburgh, EH8 9AG UK

**Keywords:** Bowel, Colorectal, Screening, Risk-stratified, Feasibility, Acceptability

## Abstract

**Purpose:**

In this scoping review, we examined the international literature on risk-stratified bowel screening to develop recommendations for future research, practice and policy.

**Methods:**

Six electronic databases were searched from inception to 18 October 2021: Medline, Embase, PsycINFO, CINAHL, Cochrane Database of Systematic Reviews and Cochrane Central Register of Controlled Trials. Forward and backwards citation searches were also undertaken. All relevant literature were included.

**Results:**

After de-deduplication, 3,629 records remained. 3,416 were excluded at the title/abstract screening stage. A further 111 were excluded at full-text screening stage. In total, 102 unique studies were included. Results showed that risk-stratified bowel screening programmes can potentially improve diagnostic performance, but there is a lack of information on longer-term outcomes. Risk models do appear to show promise in refining existing risk stratification guidelines but most were not externally validated and less than half achieved good discriminatory power. Risk assessment tools in primary care have the potential for high levels of acceptability and uptake, and therefore, could form an important component of future risk-stratified bowel screening programmes, but sometimes the screening recommendations were not adhered to by the patient or healthcare provider. The review identified important knowledge gaps, most notably in the area of organisation of screening services due to few pilots, and what risk stratification might mean for inequalities.

**Conclusion:**

We recommend that future research focuses on what organisational challenges risk-stratified bowel screening may face and a consideration of inequalities in any changes to organised bowel screening programmes.

**Supplementary Information:**

The online version contains supplementary material available at 10.1007/s10552-022-01568-9.

## Introduction

According to the World Health Organisation (WHO), colorectal cancer (CRC) is the third most common cancer worldwide with 1.80 million cases resulting in 862,000 deaths in 2018 [1]. Screening programmes can be effective in reducing the number of deaths attributed to cancer through early detection. However, a national audit found that only 58% of people in England, United Kingdom (UK), completed bowel screening and only 10% of all CRC patients are diagnosed through bowel screening [2]. Inequalities in bowel screening uptake are consistently demonstrated: participation is typically lower among those with low socio-economic status (SES) [3–5]. The COVID-19 pandemic has potentially exacerbated these inequalities in uptake, with reduced access to screening. New innovations such as stratified screening may make screening more efficient, and better able to deal with increasing colonoscopy demands.

There have been growing calls for cancer screening programmes, including bowel screening, to be risk-stratified [6], moving away from a ‘one size fits all’ approach to a more personalised one. The premise of risk stratification is that having more precise knowledge about one’s risk of CRC can be used to determine which screening modality and intensity (type of test, when screening should start/finish, frequency) should be offered to patients with varying levels of risk. Higher-risk individuals have more to gain from screening and targeting them would potentially be a more efficient and cost-effective approach. This would, however, require significant change and investment [7]; for example, screening hubs would need to adapt their IT systems to accommodate different screening regimes for different groups. With questions over ethical, legal and social implications of risk-stratified cancer screening [8], screening participants and their healthcare providers (HCPs) would need to find this approach acceptable, and the information needs of patients, in understanding this more complex approach, would need to be addressed. At present, we do not know how feasible these changes would be. Given this limited knowledge, we carried out a scoping review which is appropriate for a field whereby there are large numbers of complex and heterogeneous studies. Arskey and O’Malley [9] present four purposes of a scoping review: to examine the extent and range of research activity; to determine the value of undertaking a full systematic review; to summarise research findings; and to identify research gaps. Our objective was to examine international evidence and identify evidence gaps relating to the feasibility and acceptability of risk-stratified approaches to bowel screening to inform future research, policy and practice. Specifically, we sought evidence on organisational aspects of risk-stratified screening, its potential to worsen health inequalities, parameters of diagnostic performance, available models and tools to risk stratify, acceptability of these approaches and evidence-based guidelines.

## Methods

The scoping review protocol is registered with the Open Science Framework [10]. We have used the PRISMA Extension for Scoping Reviews checklist [11] in the reporting of this review (Supplementary file 1).

### Inclusion/exclusion criteria

Any study, both primary and secondary, which examined risk-stratified bowel screening was eligible. We included theoretical/modelling studies developing risk scores if they had undertaken either internal or external validation. Non-English studies, those which lacked sufficient detail for data extraction, protocols, and studies which included different cancer types but lacked specific data on bowel screening, were all excluded. Studies which included patients with existing health conditions (e.g. Lynch syndrome) were also excluded as this study is about screening people who are asymptomatic.

### Search strategy

Searches were conducted on six electronic databases: Medline All, Embase and PsycINFO via OVID, CINAHL Complete via EBSCOHost, The Cochrane Database of Systematic Reviews and Cochrane Central Register of Controlled Trials. The Medline strategies are available in Supplementary file 2 and combined text word searching with database-specific indexed terms. The initial search period was from database inception to the 26 June 2020 combining search terms for three major concepts (bowel cancer, screening and risk stratification) with search filters for systematic reviews and randomised controlled trials for non-Cochrane databases. A second search combined the three major concepts with other terms of interest including feasibility, acceptability and inequalities. Supplementary searches were also conducted on: PMC Europe Grant Finder, Bielefeld Academic Search Engine (BASE) and Google Scholar to identify additional relevant studies and grey literature. Forwards and backwards citation searches were also conducted via Web of Science using studies identified after the initial search and screening phase and the entire database search was updated on 18 October 2021.

### Screening and data charting

After deduplication, title, abstract and full-text screening were undertaken against the inclusion/exclusion criteria using Covidence software. The main reviewer (JC) screened 100% and two additional reviewers (SG/OB) independently screened approximately 50% each. Conflicts were resolved through discussion. A data chart was created in Excel. Data charting was carried out primarily by JC but checked by SG/OB (25% each). No quality appraisal was undertaken for this scoping review as the aim was to summarise existing evidence on the topic to inform future research, policy and practice, not to include or exclude studies based on quality [5].

## Results

**Fig. 1:**
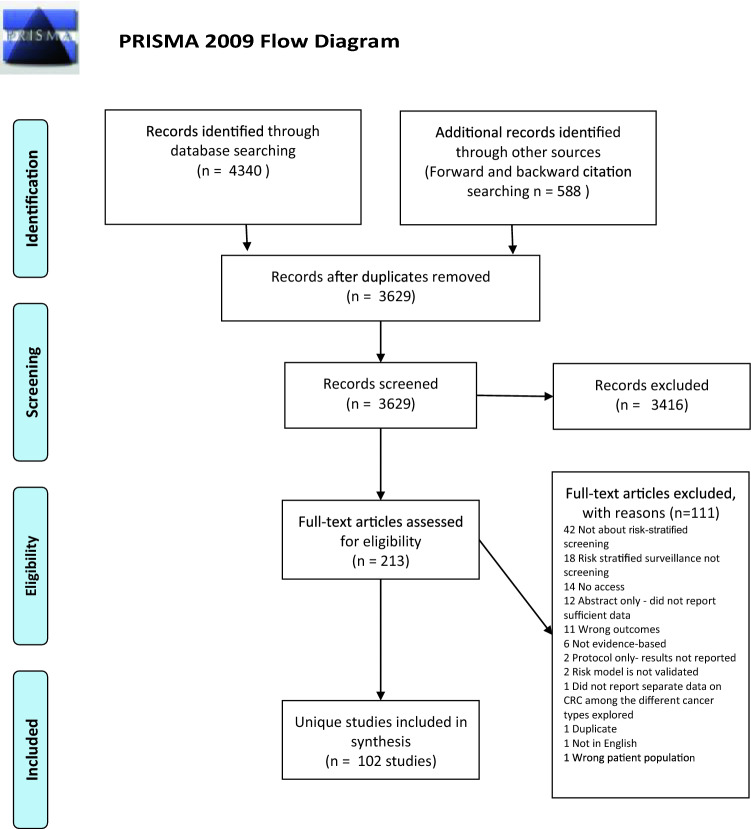
PRISMA 2009 Flow Diagram

In total, 4,340 records were identified through database searching, an additional 588 by forward and backward citation searching of initially included studies after the search bringing the total to 4,928. There were 3,629 records after duplicates were removed. These were title and abstract screened; 3,416 records were excluded at this stage. 213 records with full-texts were assessed for eligibility against the inclusion/exclusion criteria. 111 full-texts were excluded with reasons listed in the PRISMA flow diagram (Fig. 1), and 102 unique studies (some records were merged if they were part of the same study) were included in this study.

### Overview of the current state of evidence

**Fig. 2:**
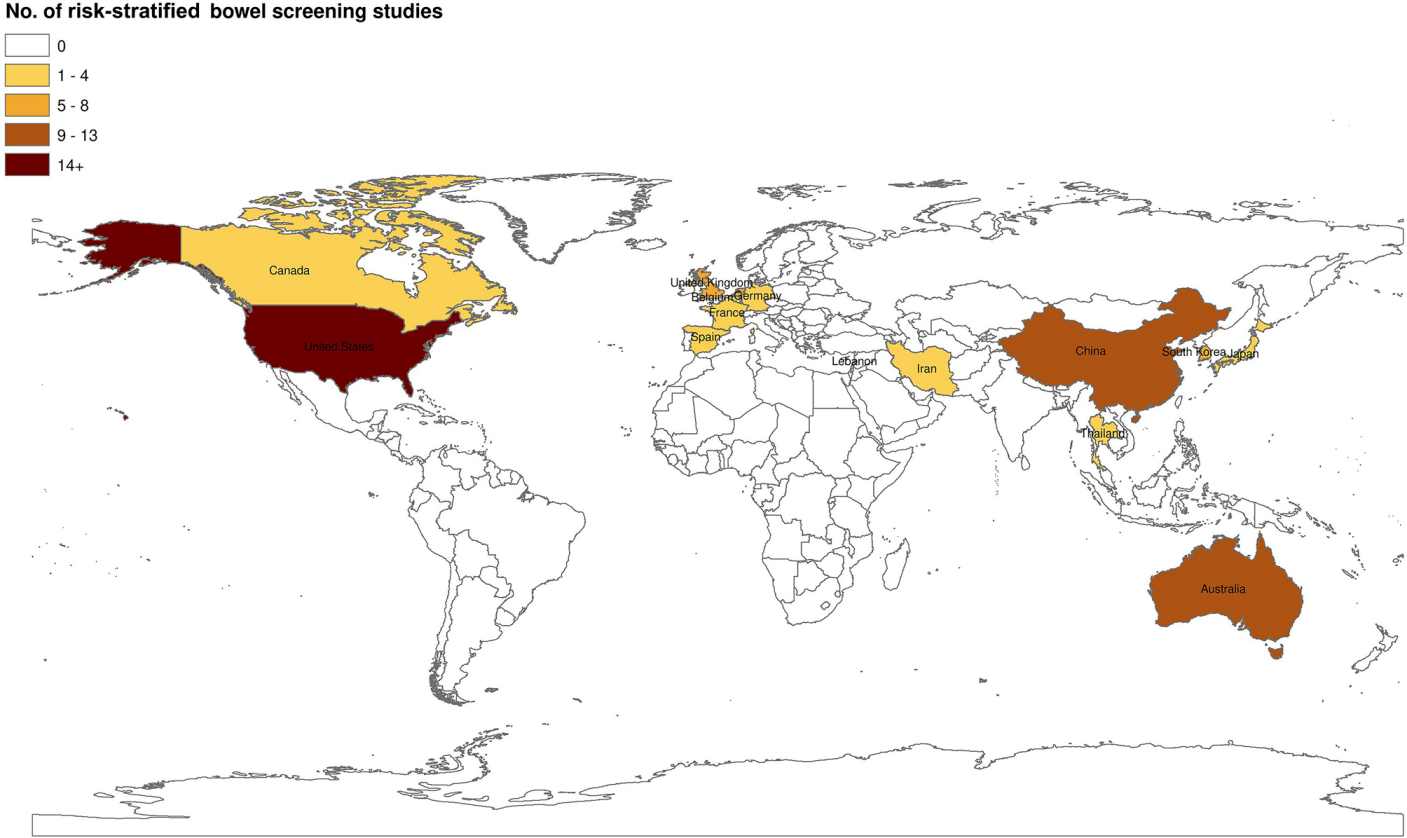
Map of included studies

Most studies were conducted in the US (*n* = 28) followed by China (*n* = 13), Australia (*n* = 11), UK (*n* = 8), Netherlands (*n* = 7), South Korea (*n* = 7), Germany (*n* = 4), Japan (*n* = 3), Thailand (*n* = 2) and one each from Canada, Belgium, France, Iran, Lebanon and Spain; 13 were multi-country studies (see Fig. 2). The studies varied in their methodological designs (Tables 1, 2, 3, 4, 5, and 6, Supplementary file 3) which ranged from primary research (mostly observational or experimental studies) (*n* = 79) to systematic (*n* = 6) and non-systematic reviews/evidence-based commentaries/editorials (*n* = 17). We did not perform a quality appraisal of the included studies as our objective was to summarise the extent and full range of evidence on the topic. We have organised the findings into the following groups: (1) the diagnostic performance of risk-stratified bowel cancer screening approaches; (2) the effectiveness of risk prediction models; (3) the use of risk prediction tools in clinical environments; (4) the acceptability of risk-based bowel screening approaches to patients and HCPs; (5) cost-effectiveness; and (6) evidence-based guidelines and recommendations for future risk-stratified bowel screening.

**Table 1:** Studies examining diagnostic performance of risk-stratified approaches

Author(s)	Study design	Eligible participants	Intervention/comparison groups	Diagnostic performance outcomes	AUC/C-statistics (*over 0.70 acceptable level)
Aniwan et al. (2015)	Feasibility trial	948 asymptomatic patients aged 50–75 in Thailand	(1) APCS (high/ moderate risk) (2) FIT ( +)/FIT (-) (3) Combined APCS and FIT: Group 1 (G1): high risk and FIT ( +) Group 2 (G2): high risk and FIT (-) Group 3 (G3): moderate risk and FIT ( +) Group 4 (G4): moderate risk and FIT (-)	*Detection rate (CRC/ACRN):* (1) CRC: 1.9%/0.3%; ACRN: 19.8%/8.0% (2) CRC: 2.2%/0.1%; ACRN: 19.6%/7.7% (3) CRC: G1 4.8%/G2 0.6%/ G3 1.0%/G4 0.0%; ACRN: G1 36.9%/G2 11.6%/G3 12.0%/G4 6.4% *PPV (CRC/ACRN):* (1) Not reported (2) CRC: 2.17% (0.81–4.67); ACRN: 19.57% (15.05–24.74) (3) Not reported *NPV (CRC/ACRN):* (1) Not reported (2) CRC: 99.85% (99.17–99.98); ACRN: 92.26% (89.98–94.17) (3) Not reported *Sensitivity (CRC/ACRN):* (1) Not reported (2) CRC: 85.71% (42.23–97.63); ACRN: 50.94% (41.05–60.78) (2) Refer to Fig. 3 in their paper *Specificity:* (1) CRC: 71.31% (68.3–74.18)/ACRN: 73.63% (70.52–76.58) (2) Refer to Fig. 3	AUC 0.86 (CRC)* AUC 0.67 (ACRN)
Chen et al. (2020)	Randomised controlled trial	Entire trial: 19,546 eligible participants aged 50–74 years from five Chinese provinces	(1) one-time colonoscopy (*n* = 3916), (2) annual FIT (*n* = 7854), (3) annual risk-adapted screening (*n* = 7776) which uses FIT and the APCS	*Diagnostic yield (CRC/ACRN) in the ITT analysis:* (1) CRC 0.23%; ACRN 2.40% (2) CRC 0.09%; ACRN 1.13% (3) CRC 0.17%; ACRN 1.66% *Detection rate (CRC/ACRN) in the as screened analysis:* (1) CRC 0.54%; ACRN 5.68% (2) CRC 0.10%; ACRN 1.25% (3) CRC 0.20%; ACRN 2.0%	Not reported
Chen et al. (2021a)	Post hoc analysis of trial arm above	Subset of 3825 (mean age 60.5), aged 50–74, who had blood samples taken were included in a separate analysis	(1) All colonoscopy participants, (2) Risk adapted screening based on lifestyle only, (3) Risk adapted screening based on PRS only, (4) Risk-adapted screening based on a combination of lifestyle and PRS	*PPV (ACRN):* (1) 10.8% (2) 13.6% (3) 23.1% (4) 31.7%	Not reported
Roos et al. (2020)	Prospective population-based trial	5979 screening naïve invitees from the Dutch FIT-based screening programme (add age)	(1) FIT only (1,952, 32.6%) (2) FIT plus an online validated family health questionnaire – FHQ—(2,379, 39.8%); (3) FHQ only (95, 1.6%). 1,553 (26.0%) neither returned the FIT or the FHQ and were classed as non-participants	*Diagnostic yield PER 1,000 (ACRN):* (1) 19.5 (16.3–23.3) (2) 19.6 (16.4–23.5) *Detection rate (CRC/ACRN):* (1) CRC 7%; ACRN 58% (2) CRC 6.5%; ACRN 54.2% *PPV (CRC/ACRN):* (1) CRC 6%; ACRN 50% (2) CRC 4%; ACRN 33%	Not reported
Chen et al. (2021b)	Cross-sectional	8592 QRA/ 11 FIT/ 20,203 Combined, aged 40–74, from the CRC screening program in China	(1) FIT only (2) Questionnaire-based risk assessment (QRA) (3) Combined FIT & QRA	*Diagnostic yield per 10,000 (ACRN):* (1) 36.8 (30.5–44.4) (2) 12.2 (8.8–16.8) (3) 46.9 (39.8–55.4) *PPV:* (1) 9.9% (8.3–11.9) (2) 1.9% (1.3–2.3) (3) 4.7% (4.0–5.6)	Not reported
Toyoshima et al. (2021)	Cross-sectional	8724 patients (mean age 53.7) who underwent colonoscopies as part of the screening programme in Japan	(1) FIT (+ / +) (2) FIT ( ±) (3) FIT (+ / +) ≥ 50 years (4) FIT ( ±) ≥ 50 years (5) FIT (+ / +) < 50 years (6) FIT ( ±) < 50 years	*Detection rate (CRC):* (1) 12.1% (2) 1.9% (3) 12.9% (4) 3.5% (5) 11.3% (6) 0.4%	Not reported
Wong et al. (2014)	Cohort	5813, aged 50–70 (mean age 57.7) from a community screening centre in Hong Kong	(1) FIT ( +) (2) FIT (-) (3) Colonoscopy (4) Combined Colonoscopy & FIT	*Detection rate (ACRN):* (1) 18% (2) 5.5% (3) 8% (4) 4.3%	Not reported
Kallenberg et al. (2016)	Post hoc analysis of the COCOS multicentre, population based RCT	1112 symptomatic participants who completed FIT and family history questionnaire	(1) FIT (2) Combined FIT with family history	*Detection rate (ACRN) at different FIT cut offs (10/15/20 µg Hb/g):* (1) 3.2%/2.7%/2.5% (2) 4.8%/ 4.4%/4.2% *Sensitivity of different cut offs (10/15/20 µg Hb/g):* (1) 36%/30%/28% (2) 52%/49%/47% *Specificity of different cut offs (10/15/20 µg Hb/g):* (1) 93%/96%/97% (2) 79%/81%/82%	Not reported
Kortlever et al. (2021)	Same as above	Same as above	(1) Risk based on age and sex-based FIT cut-off points (2) FIT only	Age and sex-based FIT cut-off concentrations necessary to achieve a uniform risk threshold for follow-up colonoscopy would range from 9.5 to 36.9 μg Hb/g in a risk model with a matched specificity to FIT with a uniform threshold of 20 μg Hb/g. Using either FIT or risk would lead to detection of ACRN in 28 of 58 individuals. Twelve individuals would be reclassified *Sensitivity:* (1) 28.7% (20.8 to 38.2) (2) 27.7% (19.9 to 37.1)	(1) AUC 0.71 (0.65–0.78) (2) AUC 0.69 (0.63–0.75)
Stegeman et al. (2014)	Same as above	Same as above	(1) Risk-based on FIT plus age, calcium intake, CRC family history and current smoking (2) FIT only	Classification improved with risk-based screening but the improvement was not significant: NRI 0.054 (p = 0.073) Sensitivity (1) 40% (2) 32%	(1) AUC 0.76* (2) AUC 0.69
Van de Veerdonk et al. (2018)	Cohort	57,421 participants who underwent a colonoscopy as part of the Flemish CRC screening programme in Belgium	(1) Age and sex-based FIT cut-off points (2) FIT only	Reference group = 56-year-old female with FIT 75 ng/ml In a 54-year-old male with 75 ng/ml the OR was 1.90 (1.84–2.14). The OR of detecting any abnormality was 32.22 (29.73–34.93) in a 74-year-old female with a FIT result of 1000 ng/ml vs. 58.43 (52.89–64.55) in male equivalent. There was a 1.2% probability of detecting CRC vs 1.6% for male equivalent. A 74-year-old female with 1000 ng/ml had a 19.7% probability of detecting CRC vs 22% for male equivalent	Not reported
Auge et al. (2014)	Cohort	3109, 50–69 (median 60) Spain	50–59 YEARS (1) 20–32 µg Hb/g (women/men) (2) 33–64 µg Hb/g (women/men) (3) 65–177 µg Hb/g (women/men) (4) > 177 µg Hb/g (women/men) 60–69 YEARS (1) 20–32 µg Hb/g (women/men) (2) 60–99, 33–64 µg Hb/g (women/men) (3) 60–69, 65–177 µg Hb/g (women/men) (4) > 177 µg Hb/g (women/men)	*Odds ratios of detecting ACRN:* 50 -59 YEARS (1) 1.15 (0.70–1.90)/2.51 (1.57–4.01) (2) Reference group/2.96 (1.83–4.76) (3) 2.35 (1.42–3.90)/3.84 (2.43–6.05) (4) 4.51 (2.70–7.56)/7.60 (4.78–12.04) 60–69 YEARS (1) 1.05 (0.63–1.72)/2.70 (1.57–4.01) (2) 1.60 (0.99–2.58)/ 3.64 (2.33–5.67) (3) 3.30 (2.07–5.24)/4.69 (2.99–7.35) (4) 4.47 (2.74–7.29)/ 11.46 (7.25–18.10) *PPV:* 50–59 YEARS (1) 23.8%/40.4% (2) 21.3%/44.4% (3) 38.8%/50.9% (4) 55%/67.3% 60–69 YEARS (1) 22.1%/42.3% (2) 30.2%/49.6% (3) 47.2%/55.9% (4) 54.7%/75.6%	AUC 0.676 (0.657–0.695)
Sekiguchi et al. (2021)	Cross-sectional	1191 40–79 (mean 63) Japan	(1) Risk score based on age, sex, CRC family history, BMI and smoking (2) Combination of risk score at 50/100/150/200 (ng Hb/mL) for 1- and 2- day FITs	*Prevalence (ACRN):* (1) Low = 3.8%; Intermediate = 9.3%; High = 17.7% (2) Not reported *PPV:* (1) 17.7% (2) 19.9%/20.3%/19.8%/20.1% (1-day FIT)/ 20.0%/20.6%/20.0%/20.3% (2-day FIT) *NPV:* (1) 92.5% (2) 93.8%/93.8%/93.5%/93.6% (1-day FIT)/ 94.4%/94.3%/93.9%/93.8% *Sensitivity:* (1) 35.7% (2) 50.0%/49.1%/46.4%/46.4% (1-day FIT)/ 56.3%/54.5%/50.0%/49.1% (2-day FIT) *Specificity:* (1) 82.8% (2) 79.1%/80.0%/80.5%/80.8% (1-day FIT)/76.6%/78.2%/79.2%/80.0% (2-day FIT)	C Statistic 0.66

**Table 2:** Systematic review studies summarising risk prediction models for risk-stratified screening

Systematic review Author	Focus of review/Review question	Search date	Search sources	Inclusion/exclusion criteria	Number of included studies	Key findings
Ma and Ladabaum (2014)	To review existing risk prediction models for colorectal neoplasia	January 1990 -March 2013	MEDLINE, Scopus, and Cochrane Library	Case control, cohort and cross-sectional studies that developed or tested risk prediction models for colorectal neoplasia for average risk populations were included. Abstracts only and non-English language articles were excluded	9 CRC risk prediction models	6 models were from the US, 1 from China, 1 from Japan and 1 from 11 Asian countries. The main risk factors included age, gender, smoking, a measure of obesity, and/or family history of CRC. 6 of the models were considered good (externally validated), 2 were fair (internally validated) and 1 was poor (unvalidated). Most of the risk prediction models have weak discriminatory power with only two (Cai et al. and Imperiale et al.) reaching the 0.70 C statistic. The majority of the models were developed among primarily White populations thus validation is required among more diverse populations to determine generalisability
Peng et al. (2018)	An overview on the development and validation of risk scores and their composition and discriminatory power for identifying people at high or low risk of AN	Until March 2018	PubMed, Embase, Web of Science	Included studies met ALL of the following criteria: 1) original research in peer reviewed journal, 2) using data from cohort, cross-sectional or RCTs to develop or validate a risk score. 3) considered at least age and sex and other risk factors, laboratory tests, genetic scores or their combination. 4) only included asymptomatic, average risk patients who underwent screening colonoscopy and 5 reported presence of AN as an outcome	22 studies evaluating 17 different risk scores	Risk scores included a median number of 5 risk factors. The most commonly considered and included factors were age, sex, FH in first-degree relatives (FDR), body mass index (BMI) and smoking; other frequently considered factors were alcohol, diabetes, NSAIDs, aspirin, physical activity, red meat and vegetable consumption, CVD and hypertension Only 7 scoring systems showed at least modest discriminatory power (AUC ≥ 0.70) in internal or external validation and meta-analysis of AUCs in 1 risk score indicated that the overall performance was relatively good
Peng et al. (2019)	Head to head validation and comparison of scores identified in Peng 2018 review against 2 large scale screening cohorts (KolosSal and BliTz)	As above	As above	As above	17 risk scores were compared: 14 from Peng 2018 and 3 additional models	Risk models used were: 6 tools from the United States, 3 tools from Korea, 2 tools from Hong Kong, 1 each from Germany, Spain, Poland, China, and Japan, and a cluster of 11 Asian cities Advanced neoplasms were detected in 1,917 (11.8%) KolosSal and 848 (11.4%) BliTz AUCs of all risk scores ranged from 0.57 to 0.65 in both studies, indicating variable, but overall modest performance in predicting presence of at least 1 advanced neoplasm
Raut et al. (2019)	To systematically review and summarise studies addessing the association of whole-blood DNA methylation markers and risk of developing CRC and its precursors	Until November 2018	PubMed and Web of Science	Not reported	19 studies reporting 102 methylation markers	5 studies in China, 3 in the US, 3 in Italy, 2 in the UK, and 1 each from Canada, Germany, Finland, Sweden, France and Lithuania. None of the risk predictions were validated in independent cohorts. AUCs were only reported for 2 studies (Heiss et al. 2017 and Nugsen et al. 2015) only two genes from the Heiss et al. 2017 study reached good discriminatory power (≥ 0.70): KIAA1549L promoters cg04036920 (0.70, p < 0.05) and cg14472551 (0.72, p < 0.05)
Stegeman (2013)	They examined to what extent the validity and performance of these cancer risk models have been evaluated	Until August 2010	Medline and Embase	Inclusion criteria were that published papers (any study design) examined multivariate risk models for breast, cervical or colon cancer (only colon analysed here). Models containing laboratory measurements were excluded	2 CRC risk prediction models	Only 2 CRC risk prediction models were identified: Freedman et al. 2009 (externally validated by Park et al. 2009) and Driver et al. 2007, both of which were based in the US. Neither of the models reached good discriminatory power. Freedman et al.'s model has C statistics of 0.610 (men) and 0.605 (women) for the model which including gastro history, medication use (aspirin/nsaid), lifestyle factors, hormone status (women only) and BMI. Driver et al.'s CRC model AUC was 0.695 for the model consisting of age, smoking, BMI and alcohol use
Usher Smith et al. (2016)	To conduct a comprehensive analysis of risk prediction tools for risk of primary colorectal cancer in asymptomatic individuals within the general population	January 2000—March 2014	Medline, EMBASE, and the Cochrane Library	Inclusion criteria: (i) primary studies published in a peer-reviewed journal; (ii) studies which identify risk factors for developing colon, rectal or colorectal cancer, or advanced colorectal neoplasia at the level of the individual; (iii) provide a measure of relative or absolute risk using a combination of two or more risk factors that allows identification of people at higher risk of colon and/or rectal cancer; and (iv) are applicable to the general population. Exclusion: Studies including only highly selected groups, or those with a previous history of colon and/or rectal cancer and conference proceedings were excluded	40 papers describing 52 risk models for inclusion in the analysis and six external validation studies	Multiple risk models exist for predicting the risk of developing colorectal cancer, colon cancer, rectal cancer, or advanced colorectal neoplasia in asymptomatic populations, and that they have the potential to identify individuals at high risk of disease. The discrimination of the models, as measured by AUROC, compare favourably with risk models used for other cancers, including breast cancer and melanoma, and several include only variables recorded in routine medical records and so could be implemented into clinical practice without the need for further data collection. Further research should focus on the feasibility and impact of incorporating such models into stratified screening programmes

**Table 3:** Studies evaluating risk assessment tools

Author(s)	Study design	Participants	Decision support tool used	Key outcomes
Harty et al. (2019)	Feasibility study	503 patients aged between 40 and 75 years old in three primary care practices covering different socio-economic areas in Melbourne, Australia	Colorectal cancer RISk Predictor (CRISP) model	The tool accurately identified patients at different levels of risk of CRC: low risk *n* = 39%, slightly increased risk *n* = 58%, moderately increased risk *n* = 2.4%. Although the majority (*n* = 424, 84%) reported the tool was easy to use, 41% were unable to complete the questions unaided
Saya et al. (2020)	Case control	4747 controls drawn from the Australasian Colorectal Cancer Family Registry	As above	Adding lifestyle and genomic risk to family history and age using simple screening algorithms would identify a larger number of people for screening who are expected to develop CRC. A personally tailored model (scenario 2) would substantially reduce the number of total screens (approximately 1.4 million fewer, a 22% decrease) but increase the number of cancers expected to occur in those unscreened (approximately 5000 more cancers over 10 years, a 24% increase)
Dezfoli et al. (2015)	Interventional study	199 patients completed the PFHQ in an intervention study. They compared this PFHQ with a ‘control’ group (186 randomly chosen patient charts) from Penn State Hershey Medical Center, US	Personal or Family History Questionnaire (PFHQ)	Clinician-led history taking was superior to questionnaire in obtaining quality patient history that can be useful for risk stratifying patients for bowel screening *Patient scores* Control group mean 1.09 (SD 1.17) Intervention group mean 0.86 (SD 1.07) P = 0.05 (difference between means) *Family history scores* Control group mean 1.45 (SD 1.86) Intervention group mean 1.24 (SD 1.9) P < 0.01 *Composite scores* Control group mean 2.54 (SD 2.27) Intervention group mean 2.09 (SD 2.32) P = 0.01
House et al. (1999)	Survey (postal)	Patients aged 30 to 69 years (mean age 44.4 years) in South West England, UK	Family history questionnaire followed by geneticist review	Risk was accurately stratified into the following groups: high (*n* = 52), intermediate (*n* = 104), low (94) and population risk (3,945). Risk was based on cumulative lifetime risk of CRC. A geneticist subsequently reviewed the risk assessment. None of the patients were reassigned to a lower risk group, only five patients who were originally assigned to the intermediate group were reassigned to the higher risk group
Naicker et al. (2013)	Cluster RCT	2000 in intervention arm (does not state *n* for control arm) aged 25–74 years in general practices in NSW and Victoria, Australia	Online family history risk tool for assisting GPs to make risk appropriate referrals for CRC screening	The tool had the ability to triage patients into appropriate family risk categories: 8% high-risk, 4.5% moderate-risk and 87.5% average-risk
Orlando et al. (2011)	Clinical trial	100 patients, 7 PCPs and 4 genetic counsellors based in two primary care practices in Greensborough, North Carolina, US	MeTree: collects personal and family health history data from patients in primary care	1. Predictive value for CRC: PPV 79% (vs 100% for PCPs) NPV 95% (vs 83% for PCPs) 2. Found to be useful in re-classifying patients for more intense screening
Orlando et al. (2014)	Hybrid implementation-effectiveness study	1184 patients (aged 18 + years old) in two primary care practices in Greensborough, North Carolina, US	As above	90% agreement in referral decision with National Comprehensive Cancer Network guidelines and 19% identification of patients for more intense screening
Rubinstein et al. (2011)	Cluster RCT	41 primary care practices in various US states (Illinois, Michigan, Ohio and Kansas). 3,283 patients aged 35–65 years	Family Healthware—delivers tailored messages for targeted cancer prevention behaviour change	Adherence to risk-based screening: 76% to 84% (intervention arm) versus 77% to 84% (control arm)
Skinner et al. (2016)	3-arm cluster RCT: CRIS with tailored information about risk and screening recommendation (*n* = 329), non-tailored CRIS (*n* = 322) and control (361)	Arm 1: *n* = 329; Arm 2: *n* = 322; Arm 3: *n* = 361 University of Texas Southwestern Medical Centre, US	Cancer Risk Intake System—collects data on demographic characteristics, personal medical and screening history, family history and concerns about screening	Screening participation was 47% (arms 1&2 combined) vs. 16% (p = 0.0001). There were differences in screening participation according to age and arms 1&2 (over 50 s showed significantly higher screening participation 53% in tailored versus 44% in non-tailored (p = 0.023))
Skinner et al. (2017)	Clinical study	2470 patients aged 25–49 years old in two primary care clinics in Dallas, US	As above	At 6-month follow up, 5.3% of those requiring colonoscopy and 13.3% of those requiring colonoscopy or FIT undertook guideline concordant screening while 6.6% received non-guideline concordant screening (FIT instead of colonoscopy) The likelihood of risk warranting screening was greater in patients aged 40–49 years (OR 2.38, CI 1.54–3.67), female (OR 1.82, 1.15–2.81), African-American (OR 1.69, CI 1.14–2.49) and non-Hispanic white (OR 2.89, CI 1.49–5.61) compared to Hispanics
Skinner et al. (2019)	Clinical study	699 out of 924 patients aged 50–75 years old in two primary care clinics in Dallas, US	As above	79.1% of elevated risk patients received screening orders (compared to 89.1% average risk), but only 44.1% received guideline concordant screening, and less than half of these completed colonoscopy
Yen et al. (2021)	RCT	229 primary care patients aged between 50–75 years at Stanford Health primary care clinics, US	(1) National Cancer Institute Colorectal Cancer Risk Assessment Tool (CCRAT); (2) Education control	At 12-month follow up, 38.9% in the CCRAT group vs 44% in the control group completed CRC screening but this was not statistically significant (OR 0.81, 0.48–1.38)
Ladabaum et al. (2016)	Prospective observational study	509 (50% women, median age 58, 61% white, 5% black 10% Hispanic, 24% Asian) patients undergoing screening colonoscopy at Stanford Hospital and Clinics, US	CCRAT	Evaluation of whether the CCRAT could accurately predict ACRN prevalence in a diverse population 11% had ACRN, 27% had nonadvanced neoplasia. Race/ethnicity distributions were similar between participants with and without ACRN. Individuals with ACRN had statistically significantly higher 10-year predicted CRC risk scores compared with those who did not have ACRN (median, 1.38 [IQR, 0.90–1.87] vs1.02 [IQR, 0.62–1.57]; P 5 .003) Prevalence of ACRN: 6% in the first or lowest quintile,8% in the second quintile, 12% in the third quintile, 15% in the fourth quintile, and 17% in the fifth or highest quintile (Cochran-Armitage trend test; P 5 .002. The odds ratio for the fifth quintile compared with the first quintile demonstrated an approximate threefold elevation in risk of ACRN (3.20; 95% CI, 1.21–8.49)
Conran et al. (2021)	Clinical study	281 primary care patients aged 40–70 years old located via the Genomic Health Initiative database at NorthShore University HealthSystem in Evanston, IL, US	Genetic Risk Scores and Family History tools (Excluded breast & prostate data)	56.9% of patients had a low GRS for CRC while 37% had an average risk and 6.1% had a high risk of CRC. Based on these risk results, younger patients were more likely to change their screening behaviour (mean 56.4 years). Those who were open to being screened more frequently was significant compared to those who planned to undergo cancer screening with the same, or less, frequency. Those with a high risk of GRS reported significantly more anxiety, as well as worrying about developing cancer
Dolatkhah et al. (2020)	Evaluation study	15 people aged 40–60 years, 5 medical oncologists, 3 gastroenterologists, 2 epidemiologists, Iran	Persian Risk Assessment Tool	Content validity: Based on experts’ opinions, the acceptable CVR was 0.40–1. Items that had a CVR < 0.62 were removed according to the Lawshe guideline, and the CVI was calculated as 0.70–1. Moreover, the mean CVR and CVI values were 0.62 and 0.93, respectively. For face validity, the risk assessment questionnaire was checked by 15 individuals, two of which were modified based on their input
Schroy et al. (2012)	Survey (self-administered)	3317 asymptomatic, average risk patients aged 50 -79 years from Boston Medical Center (endoscopy unit) or the Endoscopy Center at Brookline, US	Your Disease Risk (YDR)	Detection of ACRN: YDR RR scores were an independent determinant of ACRN (OR 1.23 per 1.0 increase in the RR score, 1.02–1.49, p = 0.033); however, when broken down into RR category only 2 categories were significantly more likely to have ACRN (much above average and very much below average). Therefore, the YDR index lacks accuracy for stratifying average risk patients into low/intermediate/high risk categories

**Table 4:** Studies examining acceptability of risk-stratified approaches

Author(s)	Study design	Participants/context	Risk stratification process assessed	Key findings
Mathias et al. (2020)	Semi-structured interviews	15 patients aged 50–75 years (mean age 59.8, SD 7.4) who were not engaging with CRC screening and 15 PCPs (mean age 46.5, SD 9.3) in Indiana, US	CRC risk prediction tool based on age, gender, family history, smoking and waist circumference	Patients found the tool easy to use and 'self-explanatory' with just one patient saying it was difficult to understand the concept of pack-years PCPs were encouraging about the tool in terms of potentially saving costs by choosing cheaper and less risky screening modalities but there were concerns over the tool's accuracy, consistency with guidelines, a lack of time to use it in clinical practice
Piper et al. (2018)	Survey	1415 US veterans 60–69	Risk-stratified screening and cessation of screening in low-risk groups	28.7% were not comfortable with stopping CRC screening in low-risk individuals and 24.3% thought it was not reasonable to use CRC risk calculators to guide screening decisions
Schroy et al. (2016)	RCT	Two arms: (1) Decision aid (*n* = 168); (2) Decision aid plus risk assessment (*n* = 173), Boston Medical Center, US	Risk index comprising 6 factors: age, sex, ethnicity, smoking, alcohol consumption, use of non-steroidal anti-inflammatory drugs	Patient preferences significantly differed according to high/low risk in arm 2. Providers perceived risk stratification to be useful in their decision making but often failed to comply with patient preferences for tests other than colonoscopy, even among those deemed to be at low risk of ACRN
Schroy et al. (2015)	Mixed-methods (interviews and survey)	9 PCPs (interviews) & 57 (survey), Boston Medical Center, US	Risk stratification in PCP decision-making preferences for average risk patients	Risk stratification perceived to be important. Few PCPs considered risk factors other than age for average risk patients. PCPs receptive to using an electronic risk assessment tool—97% said they would use often or sometimes in recommending appropriate screening tests
Van Erkelens et al. (2018)	Survey (online)	250 participants aged 61–75 years who were invited to undertake a colonoscopy due to a positive FIT result at two teaching hospitals in the Netherlands	Online family risk assessment for ‘FIT-positive’ individuals	177 (61%) did the assessment and 153 (51%) a 2-week follow-up. 91% were satisfied with the online test Anxiety scores lower at two-week follow-up for those classified as having population level risk
Walker et al. (2017)	Simulated consultations with actor patients	Fourteen GPs, nine practice nurses and six practice managers from twelve different practices in Australia	Risk assessment within simulated consultations	Staff preferred the natural frequency icon array which showed comparative risk over time to the graph. Some GPs did not always trust/agree with the recommendations, particularly when the decision was to recommend FOBT as colonoscopy is seen as the 'gold standard'. They were more likely to recommend colonoscopy even if the patient was at average risk Lack of GP consultation time would limit the use of CRISP—practice nurses would have the capacity, time and expertise to complete it with patients instead and it could be integrated into health checks to facilitate a discussion about changing unhealthy behaviours
Solbak et al. (2018)	Cohort study	9641 participants aged 18 + in the Alberta’s Tomorrow Project, Canada	Risk profiles derived from self-reported age, family history of CRC and personal history of bowel conditions	Low adherence (< 50%) to screening among average and moderate risk groups highlights the need to explore barriers to uptake of screening across patients with different risk profiles
Saya et al. (2021)	Mixed-methods	150 patients aged 45–74 who had an appointment to see their general practitioner were approached to participate, Australia	Genomic testing- test using DNA sample collected via cheek swab	73% (95% CI: 65–80%) of participants made an informed choice about the test. Testers, compared to non-testers, were more likely to make an informed choice about the test. This study demonstrates that after succinct pre-test counselling (approximately 5–10 min), most participants attending a GP clinic were able to make an informed decision about a genomic test for CRC risk
Courtney et al. (2012)	Survey	1592 participants aged 56–88 from the Hunter Community Study, New South Wales, Australia	Postal questionnaire asking about risk-based bowel screening advice and family history of CRC	The rate of screening advice was low with approximately one-third of respondents irrespective of risk category ever receiving CRC screening advice from a healthcare provider
Steele et al. (2019)	RCT	Three arms: (1) Numerical risk group with three different letters (*n* = 100); (2) Categorical risk with three different letters (*n* = 104); (3) Control group scenario receiving letter about positive result (*n* = 104) Bowel screening programme, Scotland, UK	Various hypothetical risk-based scenarios	All participants reported that they found the novel, personalised risk information materials easy-to-understand but 19.1% (arm 1) 24% (arm 2) and 29.6% (arm 3) found the information potentially distressing More than half the participants said they would still choose to have a colonoscopy even when told they are in the lowest risk group The findings show that providing all screening participants with an informed choice based on levels of risk would greatly increase demand on colonoscopy services

**Table 5:** Cost-effectiveness studies examining risk-stratified scenarios

Author(s)	Study design	Country	Risk stratification process assessed	Findings
Subramanian et al. (2017)	Microsimulation based on a previously validated model	US	Multiple (risk assessment tools in clinical practice, genetic testing, low-cost biomarker)	The personalised screening scenarios under 60% or 80% compliance are on average cost-effective, but there is large variability in the life years saved Risk-stratified screening, with the discriminatory power of 0.60, will likely not consistently result in improvements in mortality but will always result in lower harms than the present screening scenario Risk stratification approaches that cost more than $500 per person are not likely to be cost-effective even when very high levels of accuracy of 90% can be achieved If risk stratification increases compliance—especially among those at medium, increased, or high-risk— then a high-cost test can be highly cost-effective False positives are reduced by more than 48.6%, and perforations are reduced by at least 9.9%
Erenay et al. (2014)	Partially Observable Markov Decision Process (POMDP)	US	Gender, individual lesion risk, personal history of CRC and polyp based on colonoscopy results	Optimal policies reduce lifetime CRC risk and mortality and are associated with higher total quality adjusted life years (QALYs) The optimal policies suggest slightly less frequent screening for low- and high-risk females and more frequent screening for post-CRC females than males in the corresponding risk levels. Moreover, the optimal policies suggest that females stop screening later than males
Thomas et al. (2021)	Microsimulation model in cancer of the bowel (MiMiC-Bowel)	UK	Phenotypic and genetic risk	Stratified screening in which individuals are invited to screening based on personalised risk, assessed through genetic and/or phenotypic risk scores rather than age alone, is likely to save costs and reduce CRC incidence and mortality without significantly increasing resource use. The maximum that can be spent on risk assessment to be considered cost-effective is £114 per person Risk-stratified screening benefits men more than women
Sekiguchi et al. (2020)	Monte Carlo simulation model using state transition Markov	Japan	Modified version of the APCS using 8-point risk score based on sex, CRC family history, BMI and smoking	With the sufficiently good and same uptake rates (60%) for all tests (scenario 1), a strategy using colonoscopy (strategy 1) was the most effective (with the lowest CRC mortality and incidence) and cost-effective in this study. The results of the probabilistic sensitivity analysis and analysis with a high colonoscopy cost further supported the favourable effectiveness and cost-effectiveness of a strategy using screening colonoscopy
Cenin et al. (2020)	MISCAN-Colon	Netherlands	Polygenic risk scores and family history	Uniform CRC screening (compared to no screening) reduced CRC incidence by 22–69% and mortality by 35–79%. Personalised CRC screening reduced CRC incidence by 4–68% and mortality by 5–79%. Both scenarios led to a similar yield in QALYs: 0.11–0.32% more QALYs for uniform versus 0.02–0.32% personalised. But personalised CRC screening cost more due to the cost of determining risk

**Table 6:** Studies examining risk-stratified guidelines and evidence-based recommendations

Author(s)	Study design	Country	Recommendations
Avital et al. (2013)	Evidence-based guidelines	US	Race, SES and family history are important for future bowel screening risk stratification research
Jenkins et al. (2018)	Literature review	Australia	Separates screening guidance into the following categories: (1) Average-risk recommended screening every two years by iFOBT age 50–74 years; (2) moderate-risk due to family history recommended biennial iFOBT screening from age 40–49 years then colonoscopy every five years from age 50–74 years; (3) High-risk recommended biennial iFOBT from age 35–44 years then colonoscopy every five years from age 45 to 74 years
Geneve et al. (2019)	Commentary	US	Ethnicity should be included in risk-stratified bowel screening guidelines
Parkin et al. (2018)	Evidence-based guidelines	US	Individuals with a family history of CRC will need to start screening at an earlier age on the basis of category of risk
Imperiale and Monahan (2020)	Literature review	US	Future research should focus on validation of risk prediction models, conducting impact analyses via RCTs, and seek to understand patient/provider attitudes toward risk prediction models and how such tools are able to be integrated into health care systems
Sung et al. (2015)	Delphi study	Multi-country (14 Asian countries)	A risk-stratified scoring system is recommended for selecting high-risk patients for colonoscopy
Tejpar (2005)	Commentary	Belgium	Recommends early bowel screening for those with an elevated risk of CRC due to family history
Zali et al. (2016)	Mixed-methods	Multi-country (Canada, Australia and US)	Screening guidelines needs to be implemented into clinical practice to provide patient-specific advice on risk-based bowel screening
Bortniker and Anderson (2015)	Literature review	US	Current models have made some progress in discriminating high-risk groups, but work remains to be done to improve to improve the validity of them
Cooper et al. (2016)	Literature review	UK	Risk scoring systems based on a combination of FIT and other risk factors have been shown to improve the sensitivity of the predictive model
Huang et al. (2017)	Commentary	China	Four recommendations: (1) The discriminatory capacity of predictive models needs to be enhanced and externally validated; (2) The development of affordable non-invasive biomarkers should be an important focus; (3) In order for risk-based screening to be efficient, the effectiveness and sustainability of health education about the various risk factors for CRC should be enhanced in order to heighten community awareness. Acceptability, perception, attitude, and satisfaction of risk-based screening should also be evaluated; (4) Cost-effectiveness analyses are needed in different settings
Hull (2020)	Commentary	Multi-country	Five research priorities: (1) external validation of CRC risk prediction models; (2) evaluate risk prediction models on clinical decision-making and patient outcomes in multiple settings; (3) acceptability and feasibility of risk-stratified approaches to patents and healthcare practitioners; (4) modelling of optimal service delivery for screening and surveillance; (5) Artificial Intelligence and machine learning is needed to link large datasets to derive clinically useful prediction models
Lansdorp-Vogelaar (2021)	Literature review	Multi-country	Future research should investigate acceptability of risk-stratified screening as well as impact on costs and organisation. ‘Low hanging fruit’ include basing risk stratification on readily available information e.g. FIT. IT systems will need to be developed in a modular way
Lin (2012)	Literature review	Multi-country	Family history should be considered for more ‘aggressive’ screening regimes as there is a wealth of evidence on this and it appears to be cost-effective. Compliance with current guidelines is sub-optimal and may be affected by under-reporting
Wong et al. (2015)	Literature review	Multi-country	Future research should focus on external validation of the existing scoring systems, especially among populations with different characteristics. Current risk scoring systems could be refined by including genomics and other biomarkers such as genetic risk scores calculated using SNPs
Cenin et al. (2017)	Literature review	Australia	Evidence suggests that a risk-stratified approached which incorporate family history, age, gender, lifestyle, socioeconomic status and genetic profiling could improve CRC risk prediction
Fletcher (2008)	Commentary	US	Expert groups recommend that family history should be taken into account when choosing the age at which screening begins, the screening test, and the interval between tests. However, these recommendations are based on relatively weak evidence. In any case, family history of colorectal cancer is often not recorded in the medical record nor used in screening decisions

### Diagnostic performance of risk-stratified bowel cancer screening approaches

Thirteen studies [12–27] examined diagnostic performance of risk-stratified approaches to bowel screening in comparison to the Faecal Immunochemical Test (FIT). Various outcome measures of diagnostic performance were used including diagnostic yield, detection rate/prevalence, odds ratios, positive predictive values (PPV), negative predictive values (NPV), sensitivity and specificity. Only five reported discriminatory power, ranging from 0.676 to 0.86 AUC (Table 1).

An ongoing randomised controlled trial (RCT) study conducted in China [12–14] found that its risk-adapted approach based on the Asia Pacific Colorectal Scoring System (APCS) had a high participation rate and superior diagnostic yield of colorectal cancer (CRC)/advanced colorectal neoplasia (ACRN) compared to FIT but inferior yield to colonoscopy. For some sub-groups (e.g. men or 60–74-year-olds), risk-adapted screening showed a similar detection rate to colonoscopy. A post-hoc analysis of one arm of the trial examined risk-based screening based on lifestyle and polygenic risk score (PRS) and found a larger PPV (ACRN) for the combined approach when compared to either colonoscopy, lifestyle or PRS only showing a cumulative effect. A feasibility trial conducted in Thailand [15] found greater detection rate of ACRN using the APCS in combination with FIT (6.15-fold, 3.72–10.17 in the high risk with positive FIT group) although the study used a lower-than-usual threshold for FIT positivity (50 ng/mL) which may have resulted in a higher number of false positives (1 in 7 cancers were still missed). A population-based trial in the Netherlands [16–18] further identified participants who had either a positive FIT and/or positive family health questionnaire (FHQ) result, confirmed after genetic counselling, and referred them for a colonoscopy. There was no increased diagnostic yield for the combined FIT and FHQ approach, and it had a high false-positive rate (35%). Participants who returned the FHQ tended to be younger, and had higher SES, possibly due to costs of genetic testing. A similar study [19] compared FIT with a questionnaire-based risk assessment (QRA) and found that FIT was superior to the QRA or combined FIT and QRA approach. However, another study [20] found an increased detection rate of the combined FIT and FHQ when adjusting the FIT cut off points (10/15/20 µg Hb/g). A few other studies also looked at the impact of changing the FIT cut-off but instead of using family history they adjusted according to age/sex. For instance, a Spanish cohort study [21] found higher odds of detecting ACRN for men than women and when combined with faecal haemoglobin concentration levels, the risk of ACRN increased 11.46-fold amongst individuals in the highest versus those in the lowest risk category. Similar results were found by a cohort study conducted in Belgium [22] indicating that FIT may be an effective tool not only as a screening modality but also for risk stratification. However, another study using data from the Colonoscopy or Colonography for Screening (COCOS) Netherlands trial [23] found no statistically significant differences between different FIT cut-offs and matched positivity thresholds. The absolute differences between sensitivities were higher at lower FIT cut-offs, suggesting that models using age and sex may have greater benefit at low positivity thresholds. A Chinese cohort study [24] found that prior negative FIT results could be used as a risk stratification tool since detection of ACRN was greater than the combined colonoscopy and FIT group but inferior to colonoscopy alone. A Japanese cross-sectional study [25] also examined the role of FIT as a risk stratification tool, this time in combination with age, and found higher detection of CRC for 2-day FIT positive aged 50 years and over. They showed that 2-day FIT had a higher yield than one positive FIT result. Therefore, it is proposed that a 2-day FIT could help to prioritise patients for colonoscopy. Another Japanese study [26] evaluated the performance of an 8-point risk score based on age, sex, CRC family history, BMI and smoking and in combination with FIT at different thresholds for 1 and 2 days. PPV was higher in the combined risk score and FIT group with increased sensitivity but lower specificity. Lastly, a cross-sectional study conducted in the Netherlands [27] found that a risk-based model (age, CRC family history, smoking, BMI, regular aspirin use/nonsteroidal anti-inflammatory drug use, total calcium intake and physical activity) had better discrimination in distinguishing ACRN and greater sensitivity compared to FIT alone. They found that with the risk-based screening the same number of colonoscopies would lead to the detection of five more cases of ACRN, thus this combined approach has better accuracy than FIT alone and may help to reduce the number of colonoscopies required.

Overall, it is difficult to draw definitive conclusions about the efficacy of the risk-based screening approaches in comparison/combination with FIT since the results were mixed. However, diagnostic performance did improve in some studies which show promise for risk-adapted bowel screening and may help to prioritise colonoscopies for those at highest risk. Review findings suggest models based on more than just family history lead to a better detection of ACRN when used in conjunction with FIT.

### Risk prediction model validation studies

Thirty-five studies [28–62] examined the detection of CRC, ACRN or advanced proximal neoplasia by modelling various risk prediction scoring systems (Supplementary file 3). Of the 35 risk prediction models, 15 achieved good discriminatory power (AUC/C-statistic ≥ 0.70) while 11 were externally validated. The studies used a variety of risk models, most notably the APCS, originally developed in 14 Asian sites [62] but was externally validated outside of Asia [32]. The APCS was adapted by some studies, such as Korean version [42]. Additionally, risk scoring systems comprising factors such as age, gender, lifestyle factors, and polygenic risk scores were evaluated. There are too many to summarise here but many of them have been summarised in previous systematic reviews, detailed in Table 2. These reviews synthesised various risk scoring systems based on socio-demographics (age/sex), lifestyle (smoking, obesity/BMI), medication use, family history, and biomarkers. They typically found that the models had modest performance in predicting ACRN.

In summary, there is a wealth of studies examining a broad range of risk prediction models that could be used to stratify risk as part of bowel screening programmes but most models do not have an acceptable level of discriminatory power while others need to be externally validated, particularly in more ethnically diverse populations. This should be the focus of future studies looking at ways to stratify risk.

### Studies evaluating risk assessment tools in clinical practice

Sixteen studies [63–82], of various study designs, examined the clinical utility of risk stratification tools to accurately classify patients into risk groups for various cancers based on personal and family history provide recommendations for type of guidance-concordant screening and promote adherence. Eleven tools were identified in total: Colorectal cancer RISk Predictor (CRISP) [63–65]; MeTree [66–68]; Family Healthware [69]; Cancer Risk Intake System (CRIS) [70–72]; an online family history tool [73–75]; National Cancer Institute Colorectal Cancer Risk Assessment Tool (CCRAT) [76, 77]; Personal or Family History Questionnaire [78]; family history questionnaire followed by a geneticist review [79]; Your Disease Risk [80]; Persian risk assessment [81]; genetic risk score and family history tools [82]. Apart from five studies [64, 65, 73–75, 79], the rest were US-based.

These tools (Table 3), were evaluated for their ability to accurately predict the presence of CRC when a referral is made [67, 68], utility and accuracy in assigning patients to risk categories or re-classify/refine previous estimates of risk categories [63, 64, 66, 73–75, 79], concordance with existing referral guidance [71, 72, 80] and impact on screening participation [69–72].

The studies typically found the tools to be helpful in assisting with referrals, albeit with mixed evidence on whether they had improved sensitivity and specificity when compared with referral decisions based on existing practice. Utility in assigning patients to risk categories as a basis for more- or less-intense screening, or in refining categories based on less detailed information was typically reported. The accuracy of these risk assignments was assessed in several ways, including comparisons with clinical records [78] and the opinion of clinicians [79, 81]. Overall, the tools examined showed high concordance with existing guidance (that is, similar numbers of patients, with similar characteristics, would have been referred), but ability to achieve compliance with screening recommendations, in the absence of an organised programme, was less encouraging [72, 80]. While improved levels of uptake were achievable [69], the ability of participants to complete the tools without assistance was questioned in some of the studies [63, 64].

Authors of the studies raised concerns around a few issues, including comprehension of the tools by patients, potential to increase referrals and overwhelm diagnostic services, inappropriate assignation to a lower-intensity screening regime and burden of completion of the tools, for patients and HCPs. Concerns were also raised about the quality of information used to inform risk stratification; family history is not always well-recorded, and self-reports may be inaccurate [83]. Indeed, one study [78] showed that clinician-led history taking was superior to a self-administered family/personal history questionnaire. Nevertheless, overall, these risk assessment tools showed improvements in either stratification of risk based on personal or family history and, in some cases, bowel screening uptake. Future studies examining the clinical utility of risk assessment tools should consider ways in which they can be easily integrated into routine practice.

### Studies examining acceptability of risk-stratified screening to patients and providers

The principal focus of ten included studies [83–93] was attitudes towards, and acceptability of, risk-based screening. They are summarised in Table 4.

Risk-stratified approaches had variable levels of acceptability among study participants. Discomfort with being assigned to a less-intensive screening regime featured [84], mediated by factors such as trust in the treating physician, belief in the efficacy of screening and perceived threat from CRC. One study noted that HCPs were typically supportive of risk assessment tools to inform decision-making [85], but did not necessarily agree with the decision as colonoscopy was seen as the ‘gold standard’. This is an important caveat for implementing these approaches. Concerns were also sometimes expressed over the extra burden, in terms of workload and time, risk-based strategies could entail. In general, there is a preference for systems which can readily be accommodated within routine clinical practice [86, 87] as well as HCPs questioning the clinical accuracy of the tool [88]. Similarly, patients will not necessarily comply with risk-based recommendations, particularly if they are at odds with their screening preferences [89] even if it does enable them to make a more informed decision [90]. There is mixed evidence that receipt of information about higher CRC risk can lead to increased anxiety. For instance, an online risk assessment test in the Netherlands [91] did not increase anxiety levels following receipt of risk information and because it was able to acquire novel family history information in 40% of participants the authors recommend using the test in bowel screening. However, an RCT [92] conducted in Scotland, UK, found that the personalised CRC risk information was easy to understand, but the information was distressing for some. They also found that intention to undergo colonoscopy was greatest amongst the highest risk groups but even the lowest risk group showed that over 50% would undergo colonoscopy. Therefore, regardless of level of risk, the results show that two-thirds would opt for colonoscopy, increasing demand on existing services. Promisingly, a study [93] conducted in Canada showed that adherence to risk-stratified screening guidelines increased with CRC risk but the authors call for future research to address low adherence among average and moderate risk groups. However, another study [83] found that in Australia the rate of screening advice ever received was low (only a third) which suggest that more could be done to communicate risk between patient and HCP.

### Cost-effectiveness studies examining risk-stratified scenarios

Five studies [94–98] examined the cost-effectiveness of risk-stratified bowel screening. Two US studies [94, 95] showed that even though optimal risk-stratified bowel screening may not be cost-effective, they are associated with reduced CRC mortality and higher total quality adjusted life years (QALYs). False positives were reduced by more than 48.6% and perforations were reduced by at least 9.9% in one study [94] while in another study optimal policies suggest that females will undergo less frequent screening compared to males with corresponding risk levels [95]. Findings from a UK-based study [96] suggest that risk-stratified screening based on genetic and/or phenotypic risk scores as opposed to age alone are likely to save costs and reduce CRC incidence and mortality without significantly increasing resource use provided that risk assessment is kept to £114 per person. According to this study, risk-stratified screening is likely to benefit men more than women. A study in Japan [97] evaluated three screening strategies (1-Colonoscopy, 2-FIT, 3-Risk score compared to no screening) and found that colonoscopy (based on 60% uptake) was the most effective in terms of highest number of QALYS and lowest CRC incidence and deaths, however, it requires a large number of colonoscopy procedures which may put additional strain on resource use. Lastly, a study in the Netherlands [98] showed that both uniform and personalised risk-based screening led to similar yield in QALYs (0.11–0.32% versus 0.02–0.32%) but risk-based screening cost more due to the costs associated with risk stratification. On the whole, based on these modelling studies, risk-stratified bowel screening is likely to cost more while generating a similar reduction in CRC deaths and number of QALYs but these approaches are likely to reduce the burden on resource use and the frequency of screening for those deemed low risk, therefore, it may be beneficial.

### Evidence-based guidelines and recommendations for risk-stratified bowel screening

The remaining seventeen papers [99–114] examined the current national guidelines for their respective countries and/or put forward recommendations for risk-stratified bowel screening based on evidence. The US, Australia and Canada have developed evidence-based risk-stratified bowel screening guidance which are not just based on age but also personal/family history [99–102] and it is argued that such guidelines may pave the way for risk stratification in other countries. Some researchers have proposed that ethnicity should also be included in risk stratification due to the increased incidence of CRC for some groups [103]. For instance, one paper refers to the American College of Gastroenterology which recommends that bowel screening should start at age 45 (as opposed to age 50) for African Americans given that they have the highest incidence of CRC than all other ethnic groups in the US [103]. A Delphi study was conducted to update to Asian guidelines [104] on bowel screening recommended using a risk-stratified scoring system combining four risk factors (age, sex, family history and smoking status) to select patients for colonoscopy, which may help to reduce cost and workload. An evidence-based commentary by an author in Belgium [115] recommended screening those at intermediate risk due to, for instance, having a first degree relative, at an earlier age given that they have between a two- to three-fold increased risk of developing CRC. This was also suggested two other papers [105, 114] while an Australian paper recommends taking into account additional factors (age, gender, lifestyle, SES and genetic profiling) as well as family history in future risk-stratified approaches [106]. A UK-based study calls for the use of risk scoring systems in combination with FIT since some studies have shown improved sensitivity of predictive models [113]. However, there was consensus that more needs to be done to validate risk scoring systems [107–111]. Furthermore, there are calls for more research to examine the acceptability [108, 109, 112], organisational implications [108, 112] and cost-effectiveness [109] of risk-stratified bowel screening going forward.

## Discussion

The review identified important research gaps, most notably in relation to the organisation of screening services, because few studies have piloted risk-stratified approaches with most studies to date having developed models/tools to aid with risk stratification. Since adoption of risk stratification would involve profound organisational change within screening programmes, there would be constraints in terms of organisational resistance, IT infrastructure limitations and human behaviour. More research on this process of organisational change is vital if risk-stratified screening is to be introduced. Further, we identified no studies which examined the potential impact of risk-stratified approaches on health inequalities. Whilst none of the studies directly measured impact of risk stratification on health inequalities, several studies mentioned important limitations of their studies that may have salience for health inequalities. For instance, studies noted that participants tended to be from higher SES backgrounds [79] with a lack of ethnic diversity [69], higher screening adherence and greater likelihood of having medical insurance [69, 89]. One of the studies demonstrated that higher income was associated with increased risk-stratified screening compliance [93], therefore, it is possible that risk-stratified bowel screening may widen pre-existing health inequalities and this needs careful analysis. However, if we look at acceptability of risk-stratified screening for other screening programmes, it is promising to see that ethnic minority groups may look favourably on it if risk is communicated clearly and translated where necessary [116].

There are some limitations to our review. Information on risk stratification in bowel screening is difficult to categorise resulting in some overlap between the six categories we applied. Further, there were some challenges in identifying studies focused on risk-stratified screening, with some lack of clarity over what constitutes risk stratification, and outcomes of interest. Nevertheless, strengths of our study included its development according to a predefined protocol, systematic and transparent approach to identification of studies, having multiple reviewers at each stage and being reported according to the PRISMA extension for scoping reviews.

Based on the review findings, we have developed recommendations for future research, practice and policy. See Box 1.

Box 1: Recommendations for risk-stratified bowel screening (numbered in order of priority) **Research**

1. Future studies should seek to externally validate CRC risk prediction models in population-based trials to enhance generalisability to wider populations

2. Studies which include healthcare professionals’ (HCP) perspectives on the clinical relevance of risk stratification for bowel cancer screening, organisational/structural barriers (including but not limited to IT infrastructure, staff time and resources) to implementation and how these can be addressed, need further consideration

3. Health inequalities should be considered as part on any risk stratification pilot programme, especially with regards to ethnicity as the majority of risk prediction models lacked ethnic diversity

4. There is limited data on acceptability which should be more fully explored in future research before introducing a risk-stratified approach to bowel screening. Behavioural science can help with this to ensure communication of risk does not induce anxiety

**Practice/policy**

1. The implementation of risk stratification will require significant change to healthcare. HCPs need to find the approach acceptable and not burdensome. It is advised that risk assessment tools used to inform risk stratified bowel screening should be incorporated into routine clinical practice and they should first be piloted with HCPs to ensure they have confidence in the clinical accuracy of the tools

2. From a patient perspective, to avoid any potential distress, future risk stratification needs to carefully consider how best to communicate personalised risk information to patients and the reasons why a risk-stratified approach

3. It is important that governments have a long-term view in mind when considering implementing risk stratification as cost savings may be further down the line after substantial investment into re-organising bowel screening programmes

## Conclusion

This scoping review mapped out the international literature on risk-stratified bowel screening. Despite over 20 years of studies and growing calls for risk stratification, we have found a limited number of studies which have actually piloted such an approach and there are mixed results. Risk stratification has the potential to improve diagnostic performance but introducing it in national bowel screening programmes can be a challenging process. Programmes have, on the whole, been established on an ‘average risk’ basis – that is, they offer the same screening regime to everyone in the population, unless they have familial/genetic conditions (such as Lynch Syndrome) in which case they would fall under surveillance programmes instead of screening [117]. Even with this ‘one-size-fits-all’ approach, there are enormous challenges facing bowel screening programmes. These include maintaining sufficient uptake to ensure population impact on CRC outcomes, and disparities in uptake due to ethnic differences and socio-demographic factors. Screening programmes are complex, requiring systems to identify eligible patients, invite them and follow-up non-responders, provide diagnostic and treatment services with sufficient capacity to accommodate screen-detected cancers, and quality assurance protocols to ensure the maintenance of high standards. It is little wonder then, that there are few examples of attempts to incorporate risk-stratification into these complex processes – quantifying risk in target populations and offering tailored screening regimes based on this risk introduce new demands in areas such as recruitment processes, organisational systems, IT infrastructure, patient and provider education and ethical considerations.

## Supplementary Information

Below is the link to the electronic supplementary material.

Supplementary file1 (DOCX 83 KB)

Supplementary file2 (DOCX 27 KB)

Supplementary file3 (DOCX 33 KB)

## Data Availability

Available on request.
