# Supplementary material for: A scoping review of risk-stratified bowel screening: current evidence, future directions
Source: Cancer Causes Control. 2022 Mar 20;33(5):653–85. doi: 10.1007/s10552-022-01568-9 (PMC8934381; doi:10.1007/s10552-022-01568-9)
Supplement: Supplementary file 2 — Supplementary file2 (DOCX 27 KB) [file 10552_2022_1568_MOESM2_ESM.docx]

**Supplementary File 2: Original Medline search for Systematic reviews and Randomised Controlled Trialss only**

| 1. exp Colorectal Neoplasms/ |  |
| --- | --- |
| 2. ((colon* or rectal* or colorectal* or rectum or bowel*) adj3 (cancer* or neoplasm* or carcinoma* or tumour* or tumor*)).ti,ab,kw. |  |
| 3. 1 or 2 [bowel cancer terms] |  |
| 4. exp Mass Screening/ |  |
| 5. "Early Detection of Cancer"/ |  |
| 6. screen*.ti,ab,kw. |  |
| 7. 4 or 5 or 6 [ screening terms] |  |
| 8. Risk Factors/ |  |
| 9. Risk Assessment/ |  |
| 10. Precision Medicine/ |  |
| 11. (Risk adj3 (stratif* or predict* or assess* or factor* or tool* or index*)).ti,ab,kw. |  |
| 12. ((Precision or personali?ed or individuali?ed) adj3 (health or medicine or screen*)).ti,ab,kw. |  |
| 13. ("P Health" or P-health).ti,ab,kw. |  |
| 14. Biomarkers, Tumor/ |  |
| 15. (marker* or biomarker*).ti. |  |
| 16. DNA Methylation/ |  |
| 17. Genetic Predisposition to Disease/ |  |
| 18. or/8-17 [ terms for risk stratification, general and specific) |  |
| 19. 3 and 7 and 18 [colorectal dancer AND screening AND risk/personalisation terms] |  |
| 20. review.pt. |  |
| 21. (medline or medlars or embase or pubmed or cochrane).tw,sh. |  |
| 22. (scisearch or psychinfo or psycinfo).tw,sh. |  |
| 23. (psychlit or psyclit).tw,sh. |  |
| 24. cinahl.tw,sh. |  |
| 25. ((hand adj2 search$) or (manual$ adj2 search$)).tw,sh. |  |
| 26. (electronic database$ or bibliographic database$ or computeri?ed database$ or online database$).tw,sh. |  |
| 27. (pooling or pooled or mantel haenszel).tw,sh. |  |
| 28. (peto or dersimonian or der simonian or fixed effect).tw,sh. |  |
| 29. (retraction of publication or retracted publication).pt. |  |
| 30. or/21-29 |  |
| 31. 20 and 30 |  |
| 32. meta-analysis.pt. |  |
| 33. meta-analysis.sh. |  |
| 34. (meta-analys$ or meta analys$ or metaanalys$).tw,sh. |  |
| 35. (systematic$ adj5 review$).tw,sh. |  |
| 36. (systematic$ adj5 overview$).tw,sh. |  |
| 37. (quantitativ$ adj5 review$).tw,sh. |  |
| 38. (quantitativ$ adj5 overview$).tw,sh. |  |
| 39. (quantitativ$ adj5 synthesis$).tw,sh. |  |
| 40. (methodologic$ adj5 review$).tw,sh. |  |
| 41. (methodologic$ adj5 overview$).tw,sh. |  |
| 42. (integrative research review$ or research integration).tw. |  |
| 43. ((qualitativ$ adj5 synthesis$) or (scoping adj review)).tw,sh. |  |
| 44. Systematic Review.pt. |  |
| 45. or/32-44 |  |
| 46. 31 or 45 |  |
| 47. 19 and 46 [topic search PLUS SR filter] |  |
| 48. randomized controlled trial.pt. |  |
| 49. (random$ or placebo$ or single blind$ or double blind$ or triple blind$).ti,ab. |  |
| 50. (retraction of publication or retracted publication).pt. |  |
| 51. or/48-50 |  |
| 52. (animals not humans).sh. |  |
| 53. ((comment or editorial or meta-analysis or practice-guideline or review or letter) not randomized controlled trial).pt. |  |
| 54. (random sampl$ or random digit$ or random effect$ or random survey or random regression).ti,ab. not randomized controlled trial.pt. |  |
| 55. 51 not (52 or 53 or 54) |  |
| 56. 19 and 55 [topic search PLUS RCT filter] |  |

**MEDLINE EXTRA SEARCH with no study design filter but additional concepts of interest:**

| 1. exp Colorectal Neoplasms/ |  |
| --- | --- |
| 2. ((colon* or rectal* or colorectal* or rectum or bowel*) adj3 (cancer* or neoplasm* or carcinoma* or tumour* or tumor*)).ti,ab,kw. |  |
| 3. 1 or 2 [bowel cancer terms] |  |
| 4. exp Mass Screening/ |  |
| 5. "Early Detection of Cancer"/ |  |
| 6. screen*.ti,ab,kw. |  |
| 7. 4 or 5 or 6 [screening terms] |  |
| 8. Risk Factors/ |  |
| 9. Risk Assessment/ |  |
| 10. Precision Medicine/ |  |
| 11. (Risk adj3 (stratif* or predict* or assess* or factor* or tool* or index*)).ti,ab,kw. |  |
| 12. ((Precision or personali?ed or individuali?ed) adj3 (health or medicine or screen*)).ti,ab,kw. |  |
| 13. ("P Health" or P-health).ti,ab,kw. |  |
| 14. Biomarkers, Tumor/ |  |
| 15. (marker* or biomarker*).ti. |  |
| 16. DNA Methylation/ |  |
| 17. Genetic Predisposition to Disease/ |  |
| 18. or/8-17 [ risk stratification terms] |  |
| 19. 3 and 7 and 18 [ bowel cancer AND screening AND risk stratification] |  |
| 20. Feasibility Studies/ |  |
| 21. "Patient Acceptance of Health Care"/ or exp Patient Satisfaction/ |  |
| 22. (feasib* or acceptability or acceptable or implement*).ti,ab,kw. |  |
| 23. Implementation Science/ |  |
| 24. exp *"Attitude of Health Personnel"/ |  |
| 25. Mass Screening/es, og [Ethics, Organization & Administration] |  |
| 26. Medical Records Systems, Computerized/ |  |
| 27. ethic*.ti,ab. |  |
| 28. "Social Determinants of Health"/ |  |
| 29. Psychosocial Deprivation/ |  |
| 30. Sociological Factors/ |  |
| 31. Working Poor/ |  |
| 32. Hierarchy, Social/ |  |
| 33. Socioeconomic Factors/ |  |
| 34. Social Welfare/ |  |
| 35. exp Social Class/ |  |
| 36. exp Poverty/ |  |
| 37. Income/ |  |
| 38. (disparit* or inequalit* or inequit* or equity or deprivation or gini or concentration index).mp. |  |
| 39. Health Status Disparities/ |  |
| 40. Minority Groups/ |  |
| 41. Minority Health/ |  |
| 42. exp Educational Status/ |  |
| 43. (socioeconomic or socio-economic).mp. |  |
| 44. exp "Emigrants and Immigrants"/ |  |
| 45. exp Ethnic Groups/ |  |
| 46. or/20-45 [ additional concepts of interest: feasibility, acceptability, inequalities] |  |
| 47. 19 and 46 [ bowel cancer AND screening AND risk stratification AND additional concepts] |  |
