# Supplementary material for: A scoping review of risk-stratified bowel screening: current evidence, future directions
Source: Cancer Causes Control. 2022 Mar 20;33(5):653–85. doi: 10.1007/s10552-022-01568-9 (PMC8934381; doi:10.1007/s10552-022-01568-9)
Supplement: Supplementary file 3 — Supplementary file3 (DOCX 33 KB) [file 10552_2022_1568_MOESM3_ESM.docx]

Supplementary file 3: Risk prediction model studies

| **RISK SCORE** | **Study validating risk score** | **Country** | **Type of study** | **Validation** | **Study period (year/s)** | **No of participants** | **Age years, mean/median, range** | **Sex (male %)** | **Outcome** | **AUC/C stats (95% CI)** | **Data collection/source** |
| --- | --- | --- | --- | --- | --- | --- | --- | --- | --- | --- | --- |
| APCS | Yeoh et al (2011) | Multi-Country (11 Asian cities: Bangkok, Guangzhou, Hong Kong, Jakarta, Kuala Lumpur, Manila, New Delhi, Seoul, Singapore, Taipei and Tokyo) | Cross-sectional | Internal | 2014 | 860 (derivation set) /1892 subjects (validation set) | 51 | 54 | ACRN | C statistic 0.66± 0.04 (same for both sets) | Endoscopy centres in 11 Asian cities (Bangkok, Guangzhou, Hong Kong, Jakarta, Kuala Lumpur, Manila, New Delhi, Seoul, Singapore, Taipei and Tokyo) |
|  | Li et al (2016) | China | Cohort | External | 2012-2014 | 1010 | 52, 47–59 | 55.1 | ACRN | Not reported | Questionnaires/CRC screening in the Peking Union Medical College Hospital (PUMCH) and the Beijing Friendship Hospital and Beijing Chaoyang Hospital. |
|  | Corte et al (2016) | Australia | Cohort study | External | Not reported | 645 | 57 | 46.7 | CRC | AUC 0.74 (0.68-0.80) | Questionnaires/University Hospital in Sydney |
|  | Jiang et al (2020) | China | Cohort study | External | 2017-2018 | 334 (asymptomatic)/ 308 (outpatient) | 52.3 (asymptomatic)/ 54.6 (outpatient) | 43.5 (asymptomatic)/ 54.7 (outpatient) | ACRN | Asymptomatic group: AUC 0.77 (0.682-0.858); Outpatient group: AUC 0.84 (0.784-0.903) | Hospital records |
|  | Luu et al (2021) | Korea | Cohort study | External | 2002-2014 | 12520 | 50.94 | 0.651 | ACRN/CRC | AUC 0.62 (both, no CIs provided) | Cancer Screenee Cohort Study |
| Modified APCS | Wong et al (2016) | China | Cohort | Internal | 2008-2014 | 5,899 | 57.7 | 47.1 | CRC | C statistics of all systems ranged from 0.56–0.65, with sensitivities ranging from 0.04–0.44 and specificities from 0.74–0.99 | Bowel cancer screening centre in Hong Kong |
| FIT & APCS | Jung et al (2018) | South Korea | Cohort | External | 2010-2014 | 9205 (40-49 years)/ 3215 (50 years and older) | 43.5 (40-49)/56.4 | 74.1 (40-49 years)/ 62 (50 years and older) | ACRN | Not reported | Kangbuk Samsung Health Study |
| APCS & KCS | Min et al (2021) | South Korea | Cohort | Internal & External | 2003-2012 | 49235 (training set)/ 7034 (tuning set)/ 14067 (internal validation set)/ 3561 (external validation set) | 41.6±8.3 (training set)/ 41.5±8.3 (tuning set)/ 41.6±8.3 (internal validation set)/ 51.3±9.0 (external validation set) | 69.3 (training & tuning set)/ 69.9 (internal validation set)/ 60.4 (external validation set) | ACRN | DNN model 1 versus APCS (Internal validation) AUC 0.713 (0.674-0.752) and 0.662 (0.619-0.705), p<0.001*/ DNN model 1 versus APCS (External validation) AUC 0.754 (0.719-0.790), p=0.433)/ DNN model 2 versus KCS (Internal validation) AUC 0.730 (0.693-0.767) and 0.667 (0.625-0.710), p<0.001*/ DNN model 2 versus KCS (External validation) AUC 0.765 (0.728-0.801) and 0.744 (0.707-0.780), p=0.125 | Kangbuk Samsung Health Study/Kyung Hee University Hospital at Gangdong |
| KCS | Kim et al (2015) | South Korea | Cohort | External | 2009-2011 | 1316 | 49.6 ± 9.9 | 42.3 | ACRN | AUC 0.681 (CI 0.608-0.763) | Medical records and interviews |
| SNPs | Jenkins et al (2019) | US/Canada/Australia | Case control | External | Not reported | 1181 (cases)/ 999 controls | 42.7 (cases), 30-57/40.9 (controls), 25-54 | 52.2 (cases)/47.9 (controls) | CRC | Not reported | Colon Cancer Family Registry (Cases), Medicare/Driving License files (USA), telephone subscribers (Canada), electoral rolls (Australia): Controls |
| SNPs & family history | Gafni et al (2021) | UK | Case control | External | 2006-2010 | 401006 (cases)/ 2992 (controls) | 61.45 ± 6.33 (cases)/57.28 ± 7.96 (controls), 40-79 years | 45.8 | CRC | Full lifetime risk models: AUC 0.673 (0.664-0.682) combined versus AUC 0.666 (0.657-0.675) family history only | Biobank |
| Genetic risk scores | Weigl et al (2020) | Germany | Cohort | Internal | 2005-2013 | 1043 | No mean/median, 50-79 | 61.7 | ACRN | C statistics: 0.596 (no GRS)/ 0.597 (GRS) | Questionnaire and genomic data/Begleitende Evaluierung innovativer Testverfahren zur Darmkrebsfrüherkennu (BLITZ) |
|  | Guo (2020a) | Germany | Case control | External | 2003-2016 | 3827 (cases)/ 2641 (controls) | 68 (combined cases & controls) | 60.2 (cases)/60.4 (cases) | CRC | Not reported | Self-reported & patient records |
| 7-miRNA score, ERS & PRS | Raut et al (2021) | Germany | Case control | Internal | 2016 | Discovery set: 20 (cases)/20 (controls)/Prospective set: 198 (cases)/178(controls) | Discovery set: 64.8±12.3 (cases)/64.7±12.1 (controls)/ Prospective set: 64.6±5.9 (cases)/62.2±6.6 (controls) | Discovery set: 55 (cases & control)/Prospective set: 61.6 (cases)/50 controls) | CRC | AUC for 7-miRNA score: 0.802 / AUC for ERS: 0.577(0.489-0.616)/ AUC for PRS: 0.622(0.5640.681) | Gebt dem Krebs keine Chance— Onkocheck (GEKKO)/Epidemiologische Studie zu Chancen der Verhütung, Früherkennung und optimierter Therapie chronischer Erkrankungen in der älteren B (ESTHER) |
| Age, sex, obesity | Park et al (2018) | South Korea | Cohort | Internal | 2005-2015 | 34658 | 43 ± 8.6 | 71.9 | ACRN | AUC 0.700 (0.679-0.722) | Self-administered questionnaire as part of the Kangbuk Samsung Health Study |
| Sex, age, BMI, alcohol consumption, smoking and previous FIT result | Kamonwan et al (2021) | Thailand | Cross-sectional | Internal | 2009-2010 | 1311 | 56.69±4.20, 50-65 | 30.2 | ACRN | BLR model AUC 0.774 (0.706-0.842) versus CART model AUC 0.765 (0.698-0.832)/ PLR model AUC 0.767 (0.695-0.839) versus CART model AUC 0.675 (0.599-0.751) | Self-administered questionnaires |
| Personal history & FIT | Li et al (2018) | China | Cohort | Internal | 2012 | 891199 | 65.7±4.2 (Free of CRC)/66.7±4.2 (CRC) | 47 | CRC | Combined AUC 84% (82%–86%)/ FIT alone 76% (74%–79%)/Questionnaire alone 73% (71%–76%) | Questionnaires and FIT from the Tianjin community-based CRC screening |
| Model 1 (All): FOBT result, smoking status, alcohol consumption, sex, age and family history of gastro-intestinal cancer. Model 2 (negative FOBT): smoking status, sex, age at FOBT, blood test and IBS | Cooper et al (2020) | UK | Cohort | Internal | 2009-2017 | 292059 | 66.43, 60-74 | 46.74 | CRC | C statistic 0.860 | The Health Improvement Network (THIN database) |
| Age, sex, IMD, previous bowel screening history | Cooper et al (2018) | UK | Cohort | Internal | 2014 | 1810 | 66.54 | 54.7 | CRC, advanced ademonas | AUC for the Neural Network Model: 0.686 (0.659–0.712);  AUC for theRisk-adjusted Logistic Regression Model: 0.659 (0.632–0.686);  AUC for the FIT only: 0.628 (0.600–0.656). | NHS Bowel Cancer Screening System (BCSS) |
| Joint lifestyle & environmental | Balarvarca et al (2020) | Germany | Case control | internal | 2005-2013 | 1014 | 60 (no neoplasm), 57–66/ 63 (nonadvanced adenoma), 58–68 / 64 (ACRN), 58–69 | 63.01 | ACRN, nonadvanced adenoma, no neoplasm | Joint Environmental-genetic risk score: AUC =0.64 (0.60–0.67) Environmental only: AUC= 0.584 (0.545–0.622) | BliTz study(Begleitende Evaluierung innovativer Testverfahren zurDarmkrebsfrüherkennung |
| Age, sex & family history | Lin et al (2006) | US | Cross-sectional | Internal | 2001-2004 | 1493 (validation set) | 59.6±8.1 | 49.2 | ACRN | Not reported | Virginia Mason medical center screening program |
| Age, smoking, alcohol intake, height and a combined sex/race/ethnicity | Schroy et al (2015) | US | Cross-sectional | Internal | 2205-2012 | 3543 | No mean/median, 50-79 | 50.53 | ACRN | AUC 0.69 (0.66–0.72) | Boston Medical Center and Tufts medical screening program. Questionnaires. |
| Age, gender, family history, body mass index and self-reported ischaemic heart disease | Wong et al (2015) | China | Cohort | Internal | 2008-2014 | 3789 (validation only) | 57.70 ± 4.92 (validation cohort) | 47 (validation only) | Proximal and synchronous proximal/distal neoplasia (Ademona and advanced neoplasia) | Prediction of proximal neoplasia: AUC 0.71 Prediction of synchronous proximal and distal neoplasia: AUC 0.65 | Questionnaires/Hong Kong Screening program |
| Personal CRC history, age, sex, ethnicity, BMI, smoking and health conditions (e.g. diabetes, heart disease etc) | Nartowt et al (2019) | US | Cross-sectional | Internal | 1997–2016 | 58376 to test the ANN | No mean/median, 18-85 | Not reported | CRC | AUCs 0.84 (hypertension & family history) versus 0.68 (hypertension without family history)/ AUCs 0.75 (no hypertension with family history) versus 0.58 (no hypertension and no family history) | 1997–2016 responses to the NHIS sample adult questionnaire from the Centers for Disease Control and Prevention (CDC) |
| Age, gender, CHD, egg intake and stool frequency | Chen et al (2014) | China | Cross sectional | Internal | 2011-2012 | 905 | 56.6±10.1 | 43.4 | ACRN | AUC 0.75 (0.69-0.82) | Questionnaire/Affiliated Yixing Hospital  of Jiangsu University |
| Age, BMI, smoking and alcohol use | Driver et al (2007) | US | Cohort | Internal | 1982-2004 | 21581 | 61 (CRC cases)/54 (Free from CRC) | 100 | CRC | AUC 0.695 /C Statistic =0.692 (no CIs reported) | Mailed questionnaires/Physician's Health Study |
| Age, waist circumference, occupational sitting time and diabetes | Guo (2020b) | China | Cohort | Internal | 2006-2016 | 92923 | 59 (CRC cases)/52(Free from CRC) | 100 | CRC | AUC 0.66 (0.63-0.68) | Interview with physican or nurse who administered a standardised questionnaire/Kailuan Cohort Study |
| Risk factors: age, sex, smoking, ethanol use, metabolic syndrome and red meat consumption/ Protective factors: being married/living with partner, advanced education, regular aspirin use and non-steroidal anti-inflammatory drugs, and physical activity | Imperiale et al (2021) | US | Cohort | Internal | 2004-2011 | 3025 (derivation set)/1475 (validation set) | 57.3±6.5 (derivation)/57.2±7.0 (validation), 50-80 | 48.4 (derivation set)/48.5 (validation set) | ACRN | C statistics: 0.77 (derivation)/0.78 (validation) | Mailed 50 item survey |
| Personal health conditions, family history, age, sex, ethnicity, BMI, diabetes and smoking status | Nartowt et al (2020) | US | Cross-sectional | TRIPOD2b/3 | Not reported | Various | Not reported | Not reported | CRC | Not reported | National Health Interview Survey (NHIS) datasets |
| Age, overweight/obesity, smoking and red meat consumption | Sharara et al (2020) | Lebanon | Cross sectional | Internal (bootstrapping) | 5 -year period (dates not reported) | 980 | 61 ± 8 | 48.9 | ACRN | AUC of 0.73 (0.66- 0.79, P < 0.001) | American University of Beirut Medical Center (AUBMC) -asymptomatic patients scheduled for screening colonoscopy |
| Age, sex, family history and lifestyle factors including body mass index (BMI), smoking status, alcohol, regular moderate-to-intensity physical activity | Shen et al (2021) | China | Case control | Internal | 2012-2016 | 176432 | 60.28 (training set)/ 60.15 (validation set) | 49.29 (training set)/48.50 (validation set) | CRC | AUC 0.624 (0.604–0.643) (training set)/ AUC 0.630 (0.604–0.655) (validation set) | Residents of the local Songjiang District community in Shanghai, East of China |
| Lifestyle | Vitellius et al (2021) | France | Cross-sectional | External (comparing 3 previously developed scoring systems: Betés, Aleksandrova and Kaminski) | 2013 - 2016 | 11706 | 60.0, 54-65 | 47.2 | ACRN/CRC | Betés score: AUC 0.63 (0.61–0.66) for lesions, 0.65 (0.61–0.68) for advanced neoplasia and 0.65 (0.58–0.72) for predicting screen-detected CRC/ Adapted HLI score: AUC 0.61 (0.58–0.65) for lesions, 0.61 (0.56– 0.65) for advanced neoplasia and 0.55 (0.45–0.65) for predicting screen-detected CRC/ Kaminski score: AUC 0.65 (0.63–0.68) for lesions, 0.65 (0.61–0.68) for advanced neoplasia and 0.69 (0.62–0.76) for predicting screen-detected CRC | Cap Santé 49 ( Maine and Loire screening center In France) |
| Age, sex, smoking status, body mass index, complete blood count, blood chemistry, and tumor marker | Yang et al (2021) | South Korea | Cross-sectional | Internal | 2003 - 2012 | 56269 (development group)/14067 (validation group) | 41.6 | 69.4 | ACRN | LR model AUC 0.724 (0.684-0.765)/DNN model AUC 0.760 (0.724 to 0.795), p < 0.001 | Kangbuk Samsung Hospital Health Screening Center, Seoul, Korea |
| Age, sex, family history of colorectal cancer, smoking, body mass index, serum levels of fasting glucose, low-density lipoprotein cholesterol, and carcinoembryonic antigen | Yang et al (2017) | South Korea | Cross-sectional | Internal | 2003 - 2012 | 49130 (derivation set)/21052 (validation set) | 41.6±8.3, 20–84 | 69.4 | ACRN | SCS score 0.682 (0.675-0.688) versus APCS AUC 0.653 (0.646-0.659), p=0.003/KCS AUC 0.658 (0.652-0.664), p=0.004 | Kangbuk Samsung Hospital Health Screening Center, Seoul, Korea |
| Age, sex, CRC family history, smoking, drinking, BMI, medical conditions and medication use (aspirin/nsaid) | Wong et al (2020) | Multi-country: China (2 sites including Beijing and Xi’an), Hong Kong, Japan (Tokyo), Korea (Seoul), Thailand (Bangkok), Malaysia (2 sites including Sabah and Kuala Lumpur), the Philippines (Manila), Singapore, Taiwan (2 sites including Taipei and Kaohsiung), Brunei (Bandar Seri Begawan), and Pakistan (Karachi) | Cohort | Internal | Not reported | 5303 (derivation set)/ 2651 (validation set) | 58.13±6.55 (derivation set) / 58.19±6.57 (validation set), 50-75 | 47.9 (derivation set)/ 48.5 (validation set) | APN | C statistic 0.74 (0.68-0.79) | Self-administered questionnaire |

**(Colorectal cancer = CRC, Advanced Colorectal Neoplasia = ACRN, Advanced Proximal Neoplasm = APN)**
